# Rescue From Permanent Kidney Injury in Acute Thrombosis of Both Renal Veins, the Inferior Vena Cava, and Both Iliofemoral Veins by Catheter-Based Thrombectomy

**DOI:** 10.1177/15266028241233229

**Published:** 2024-02-22

**Authors:** Felix Hofer, Jan Mueller, Dragan Copic, Sabine Eichinger-Hasenauer, Christian Kinstner, Lukas Reider, Marieke Merrelaar, Stephan Korn, Wolfgang Bauer, Renate Koppensteiner, Constantin Aschauer, Gere Sunder-Plassmann, Alice Schmidt, Oliver Schlager

**Affiliations:** 1 Division of Cardiology, Department of Medicine II, Medical University of Vienna, Vienna, Austria; 2 Division of Nephrology, Department of Medicine III, Medical University of Vienna, Vienna, Austria; 3 Division of Hematology and Hemostasis, Department of Medicine I, Medical University of Vienna, Vienna, Austria; 4 Division of Cardiovascular and Interventional Radiology, Department of Bioimaging and Image-Guided Therapy, Medical University of Vienna, Vienna, Austria; 5 Department of Emergency Medicine, Medical University of Vienna, Vienna, Austria; 6 Department of Urology, Medical University of Vienna, Vienna, Austria; 7 Department of Dermatology, Medical University of Vienna, Vienna, Austria; 8 Division of Angiology, Department of Medicine II, Medical University of Vienna, Vienna, Austria

**Keywords:** deep vein thrombosis, thrombectomy, renal vein thrombosis, venous stent

## Abstract

**Case:**

A 33-year-old man with previously diagnosed lupus membranous nephropathy presented with painful swelling in both legs. Laboratory tests revealed acute kidney injury, and imaging studies by duplex ultrasound and computed tomography scan showed acute thrombosis of both renal veins, the infrahepatic inferior vena cava, and both iliofemoral venous segments. Initially, pharmacomechanical thrombolysis led to an insufficient morphological result. The therapeutic breakthrough was achieved by catheter-based mechanical thrombectomy of the infrarenal vena cava and both renal veins, which successfully cleared all affected venous segments from thrombus, paralleled by improvement of the patient’s condition. However, after 1 week, the patient experienced recurrent thrombosis of the right renal vein with hemorrhagic infarction of the right kidney. After further optimization of immunomodulatory and antithrombotic therapy, a repeated catheter-based mechanical thrombectomy resulted in sustained clinical improvement and preservation of renal venous drainage and kidney function.

**Conclusion:**

Extensive acute thrombosis of both renal veins, the inferior vena cava, and both iliofemoral venous segments is a rare emergency potentially threatening kidney function. Immediate effective thrombus removal is essential to preserve kidney function and can be achieved by catheter-based mechanical thrombectomy embedded in a comprehensive immunomodulatory and antithrombotic therapeutic concept.

**Clinical Impact:**

This case demonstrated the efficacy of a catheter-based therapeutic approach in patients with extensive thrombosis of the venous system. A catheter-based approach must be embedded in a comprehensive medical therapeutic concept, which is essential to achieve a sustainable result.

## Case

A 33-year-old male patient with a history of systemic lupus erythematosus (SLE) and psoriasis vulgaris presented with acute painful swelling and purple discoloration of both lower limbs, as well as serologic evidence of acute kidney injury.

A month earlier, the patient had undergone a kidney biopsy because of proteinuria (urinary protein-to-creatinine ratio 2200 mg/g, serum creatinine not elevated) revealing a lupus membranous nephropathy (lupus nephritis class V). After the histological diagnosis had been established, the initiation of an immunosuppressive therapy had been planned, but not been implemented up to the patient’s current presentation.

The initial laboratory tests showed a sudden increase of his previously normal serum creatinine concentration to 2.6 mg/dL, and an acute inflammatory reaction with a C-reactive protein concentration of 18.6 mg/dL (limit <0.5 mg/dL) and a leukocyte count of 14 G/L.

**Figure 1.**
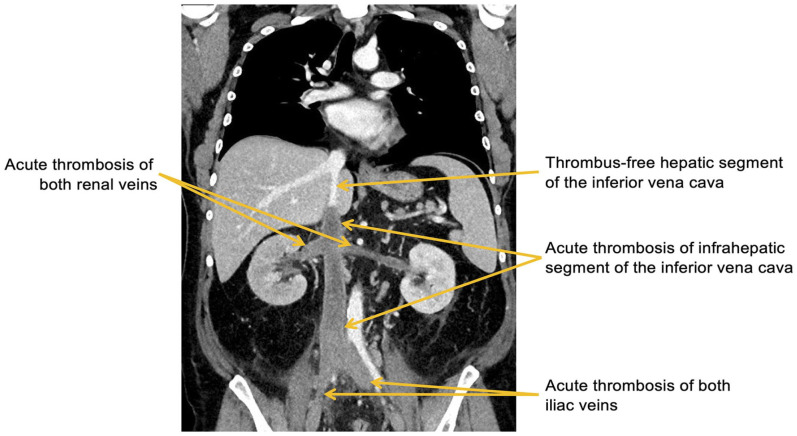
Thoracoabdominal computed tomography showing acute thrombosis of the infrahepatic inferior vena cava, both renal veins and both iliac veins.

Comprehensive imaging studies by duplex ultrasound of both lower limbs and thoracoabdominal computed tomography showed an acute thrombosis of the infrahepatic inferior vena cava, both renal veins, both iliac veins, as well as both femoral veins (Figure 1). Moreover, a hemorrhagic infarction of the right kidney as a consequence of renal vein thrombosis was found. Both popliteal veins and the intrahepatic segment of the inferior vena cava were free of thrombus.

The patient immediately received therapeutic anticoagulation therapy with low-molecular-weight heparin adjusted to his kidney function.

### First Thrombus Removal

Following a thorough review of all findings and an interdisciplinary discussion, an immediate catheter-based thrombus removal strategy was started. As adequate effective large-bore mechanical thrombectomy devices were not available at initial presentation, a pharmacomechanical thrombectomy approach was initiated to reduce the thrombus burden until delivery of a mechanical thrombectomy system: therefore, ultrasound-accelerated catheter-directed thrombolysis (EKOS, Ecosonic Endovascular system) was initiated by 2 multi-lumen sideport infusion catheters, which were inserted via both femoral veins with the treatment zones overlapping in the infrahepatic inferior vena cava. Over a duration of 12 hours, a total dose of 20 mg of recombinant tissue plasminogen activator was safely administered without any sign of bleeding. After 12 hours, a beginning improvement of serum creatinine was observed; however, no substantial reduction of thrombus mass was achieved as documented by duplex sonographic follow-up.

Immediately therefore, a mechanical catheter-based thrombectomy procedure was performed using the ClotTriever (Inari) system. Both popliteal veins were punctured, a 16-French ClotTriever sheath was inserted into the right popliteal vein, and a 13-French ClotTriever sheath was inserted in the left popliteal vein. For pulmonary protection, FlowTriever XL-Catheter disks were deployed in the intrahepatic segment of the inferior vena cava via an additional access with a 12-French sheath, which was inserted into the right jugular vein.

The thrombus passage was performed by stiff-angled glidewire from each popliteal access and support catheter was used to pass the deployed FlowTriever XL-Catheter disks. After wire-exchange by extra-stiff wires, 9 thrombectomy passes were performed via the left popliteal vein, and 4 thrombectomy passes were performed via the right popliteal vein.

**Figure 2.**
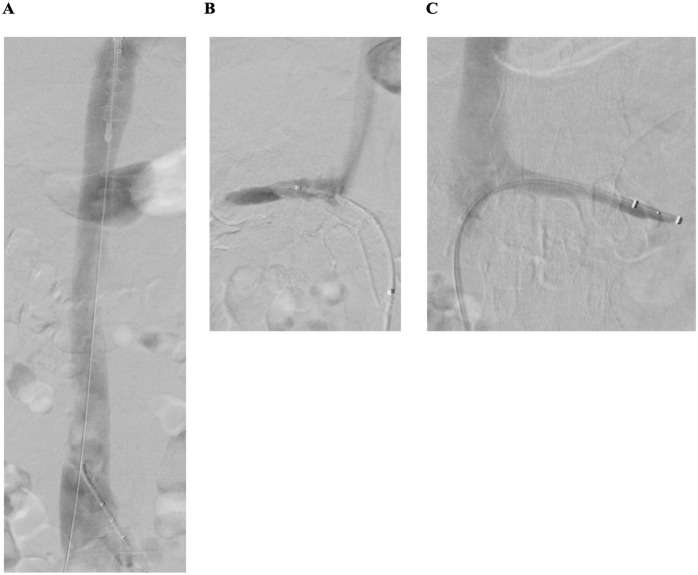
Result of catheter-based mechanical thrombectomy of the inferior vena cava, both iliofemoral venous segments, and both renal veins: (A) restored venous flow in the inferior vena cava, (B) restored flow in the right, and (C) and left renal vein after thrombectomy with Angiojet ZelanteDVT catheter (Boston Scientific) of both renal veins.

Control phlebography confirmed brisk continuous flow via both iliofemoral venous axes and the inferior vena cava, as well (Figure 2A). Subsequently, thrombectomy of both renal veins was performed using the Angiojet ZelanteDVT catheter (Boston Scientific). To additionally support venous drainage of the kidneys, prolonged balloon angioplasty of both renal veins was performed. Final phlebography showed a spontaneous contrast drainage of both renal veins (Figure 2B and C). Moreover, intravascular ultrasound (IVUS) was used to confirm adequate thrombus removal and to rule out residual venous obstructions. Furthermore, duplex ultrasound confirmed venous flow in both renal veins and in the inferior vena cava. A good flow with outflow into the inferior vena cava was observed in the duplex control, with good respiratory-modulated flow in the inferior vena cava.

### Anticoagulation and Immunosuppressive Therapy

Following mechanical catheter-based thrombectomy, an immediate reduction of leg swelling and an improvement of kidney function were observed.

**Figure 3.**
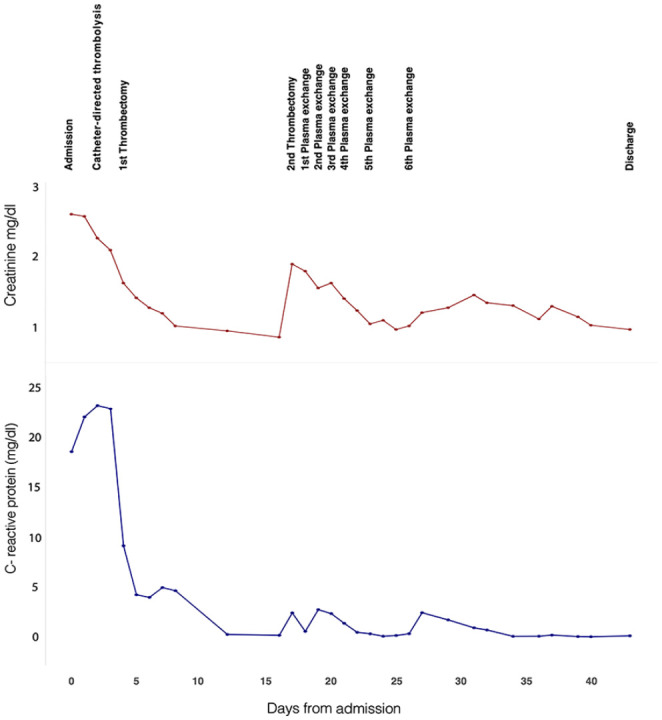
Timeline of therapeutic interventions and laboratory parameters (creatinine and C-reactive protein) levels from time of admission (day 0) to time of discharge (day 43).

The cause of thrombosis was attributed to a state of hypercoagulability due to the conjoint occurrence of the nephrotic syndrome, an inflammatory state (nephritis), and the underlying immunologic disorder. The laboratory assessment for lupus anticoagulant was negative. Therefore, an immunosuppressive triple therapy was established, consisting of prednisone, mycophenolate mofetil, and hydroxychloroquine. In this patient, a combination of therapeutic anticoagulation with low-molecular-weight heparin (enoxaparin) and anti-Xa level monitoring together with aspirin 100 mg once daily was used as postinterventional antithrombotic regime. The dosage of enoxaparin varied from 6000 IU to 10 000 IU following anti-Xa monitoring. Over the following days, 2 additional postinterventional duplex ultrasound examinations confirmed regular flow in both renal veins and in the inferior vena cava. Moreover, a stabilization of kidney function, as well as a reduction of inflammatory parameters, was observed. The timeline of therapeutic interventions and the course of serum creatinine and C-reactive protein are depicted in Figure 3.

### Recurrent Thrombosis

One week after thrombectomy, now receiving immunosuppressive treatment and therapeutic anticoagulation with low-molecular-weight heparin, the patient developed gross hematuria accompanied by recurrent acute kidney injury. Immediate imaging by duplex ultrasound and computed tomography detected a recurrent acute thrombosis of the right renal vein with hemorrhagic infarction of the right kidney, and a thrombotic lesion in the infrarenal inferior vena cava. Furthermore, gross hematuria resulted in an acute urinary retention via thrombus formation within the bladder.

### Repeated Thrombus Removal

**Figure 4.**
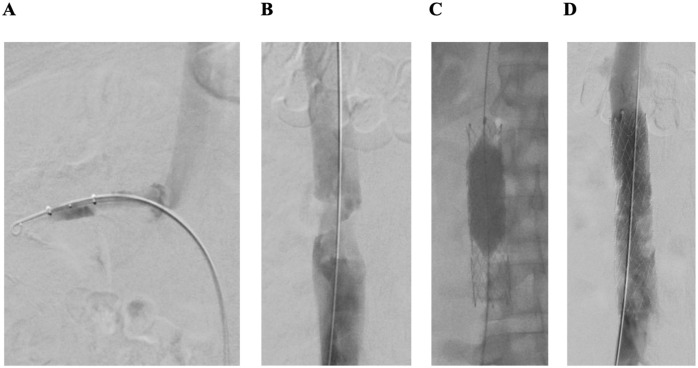
Mechanical thrombectomy of the re-occluded right renal vein and the inferior vena cava: (A) thrombectomy with Angiojet ZelanteDVT catheter (Boston Scientific) of right renal vein, (B) thrombotic stenosis of inferior vena cava, (C) postdilation after stent placement in the inferior vena cava (Abre stent 20/100 mm, Medtronic) with a non-compliant high-pressure balloon implantation (Atlas Gold 20/40 mm, BARD/BD), and (D) control phlebography of inferior vena cava after stent implantation.

As both iliofemoral venous segments were free of thrombus, the right common femoral was punctured and a mechanical thrombectomy of both renal veins and of the infrahepatic inferior vena cava was performed using Angiojet ZelanteDVT catheter (Boston Scientific, Figure 4A). For thrombectomy of the infrarenal inferior vena cava, the Angiojet ZelanteDVT catheter (Boston Scientific) was used. A subsequent IVUS revealed a residual venous obstruction of the infrarenal inferior vena cava, which subsequently was treated by balloon angioplasty with a non-compliant 20/40 mm balloon (Atlas Gold, BARD/BD) and implantation of a 20/100 mm self-expandable venous stent (Abre, Medtronic, Figure 4B and C).

Postinterventional phlebography confirmed a venous drainage of both renal veins and a satisfying flow of the inferior vena cava. Duplex ultrasound confirmed regular venous flow modulation of the inferior vena cava and of both renal veins.

### Subsequent Medical Therapy

To further escalate the immunomodulatory approach, we subsequently initiated 6 sessions of therapeutic plasma exchange in addition to the ongoing immunosuppressive therapy. According to continuation of gross hematuria, another computed tomography was performed showing diffuse hemorrhagic infarction of the right kidney with parenchymal damage and intact left kidney parenchyma. In addition, bleeding was aggravated by transient thrombocytopenia, which most likely was caused by the underlying systemic immunological disorder. After exclusion of pseudo-thrombocytopenia and heparin-induced thrombocytopenia, low-molecular-weight heparin with anti-Xa monitoring was continued and aspirin was stopped.

Following this strategy, the blood cell count stabilized, and the patient’s kidney function improved. Finally, we discharged the patient with excellent kidney function, on an immunosuppressive therapy consisting of prednisone, mycophenolate mofetil, and hydroxychloroquine together with oral anticoagulation with a vitamin K antagonist with an international normalized target of 2.0 to 3.0.

### Follow-up

The patient returned for a clinical and duplex sonographic follow-up examination after 2 months. At this time, the patient was free of symptoms, had a stable kidney function (serum creatinine 0.81 mg/dL) and duplex ultrasound confirmed regular venous flow of the inferior vena cava, of both renal veins and of both iliofemoral venous tracts.

## Discussion

This case report highlights the role of immediate catheter-based mechanical thrombectomy in patients with extensive venous thrombosis. Moreover, the course of this patient underlines the importance of embedding interventional treatment in a comprehensive medical therapeutic concept targeting potential underlying pathologies predisposing to venous thrombosis.

Especially in case of unusual locations of venous thrombosis, the role of underlying risk factors, such as a systemic autoimmune disorder, needs to be considered for establishing a holistic treatment concept. In the presented case, SLE and the associated thrombo-inflammatory state most likely contributed to the development of venous thrombosis, as well as to the occurrence of recurrent thrombosis.^1,2^ An individualized antithrombotic approach, maximal escalation of immunomodulatory therapy in combination with therapeutic plasma exchange substantially contributed to the prevention of further recurrence of venous thrombosis after repeated interventional thrombectomy.

Apart from immunological factors, the pre-existing nephrotic syndrome warrants consideration as state of hypercoagulability. There is a strong association between nephrotic syndrome and venous thromboembolism, with deep vein thrombosis and renal vein thrombosis being particularly common.^3^ In a retrospective study of 298 nephrotic patients followed for a mean period of 10 years, the annual incidence of venous thromboembolism was 1.02% per year.^4^ Renal vein thrombosis is a classic site for thrombosis in patients with nephrotic-range proteinuria, with a prevalence varying from 22% to 52%.^3,5,6^ The etiology of the hypercoagulable state in nephrotic patients is not fully understood. Evidence suggests ongoing subclinical coagulation, as demonstrated by measures of hemostasis activation such as the plasma level of fibrinopeptide A and D-dimer. Multiple hemostatic abnormalities have been described, including decreased levels of antithrombin (normal values in this case) and plasminogen due to urinary losses, increased platelet activation, hyperfibrinogenemia, inhibition of plasminogen activation, and the presence of high-molecular-weight circulating fibrinogen moieties. The possibility of immune complex injury in the glomerulus resulting in systemic effects on clotting has also been proposed.^3,7–11^ Membranous nephropathy poses the highest risk for venous thromboembolism among the causes of nephrotic syndrome, especially when associated with SLE. In a cohort study comprising 66 patients diagnosed with SLE, the incidence of venous thromboembolism was observed to be 23%, with 15 patients affected over a mean follow-up period of 6.9 years.^12^

Regarding the consideration of endovascular treatment options of acute renal vein thrombosis, recent data suggested a benefit of endovascular thrombus removal strategies in terms of higher rates of thrombus clearance and—especially in the acute phase (less than 14 days after onset)—better improvements of kidney function.^13^ In the presented case, the additional involvement of the inferior vena cava and both iliofemoral veins further aggravated the risk of persisting clinically relevant thrombotic occlusion and ongoing deterioration of kidney function. The implementation of pure mechanical thrombectomy devices in an endovascular thrombus removal strategy allows the efficient removal of large amounts of thrombus without substantially increasing the risk of bleeding. Nevertheless, the course of disease in this case underlines the necessity of a comprehensive treatment concept conjointly employing immunomodulatory therapy, antithrombotic therapy, and thrombus removal to preserve kidney function in such an extensive manifestation of venous thrombosis.

## Conclusion

Acute kidney injury due to thrombosis of both renal veins, the inferior vena cava, and both iliofemoral veins is a rare but potentially life-threatening manifestation of patients with lupus membranous nephropathy. It requires a comprehensive therapeutic concept, combining immediate thrombus removal with immunomodulatory and antithrombotic therapy to achieve thrombus clearance, prevent recurrence, and restore kidney function. This case clearly underscores the importance of an early multidisciplinary approach to tailor the therapeutic management of extensive venous thrombosis.
